# The impact of an ultrasound atlas for scoring salivary glands in primary Sjögren’s syndrome: a pilot study

**DOI:** 10.1007/s10067-023-06696-4

**Published:** 2023-08-08

**Authors:** Nanna S. Schmidt, Viktoria Fana, Mads Ammitzbøll Danielsen, Hanne M. Lindegaard, Anne Voss, Hans Christian Horn, John B. Knudsen, Keld-Erik Byg, Melanie Birger Morillon, Søren Andreas Just, Uffe M. Døhn, Lene Terslev

**Affiliations:** 1 Department of Rheumatology, Odense University Hospital, Odense, Denmark; 2 Copenhagen Center for Arthritis Research, Center for Rheumatology and Spine Diseases, Rigshospitalet, Glostrup, Denmark; 3 Department of Clinical Research, University of Southern Denmark, Odense, Denmark; 4 Department of Neurology, Odense University Hospital, Odense, Denmark; 5 Section of Rheumatology, Department of Medicine, Svendborg Hospital – Odense University Hospital, Svendborg, Denmark; 6 Department of Clinical Medicine, University of Copenhagen, Copenhagen, Denmark

**Keywords:** OMERACT, Reproducibility of results, Salivary glands, Sjögren’s syndrome, Ultrasonography

## Abstract

The objective of this pilot study was to assess the impact of a salivary gland ultrasound (SGUS) atlas for scoring parenchymal changes in Sjögren’s syndrome by assessing the reliability of the scoring system (0–3), without and with the use of the SGUS atlas. Ten participants with varying experience in SGUS contributed to the reliability exercise. Thirty SGUS images of the submandibular and parotid gland with abnormalities ranging from 0 to 3 were scored using the written definitions of the OMERACT SGUS scoring system and using the SGUS atlas based on the OMERACT scoring system. For intra-reader reliability, two rounds were performed without and with the atlas—in the 2nd round the 30 images were rearranged in random order by a physician not included in the scoring. Inter-reader reliability was also determined in both rounds. Without using the atlas, the SGUS OMERACT scoring system showed fair inter-reader reliability in round 1 (mean kappa 0.36; range 0.06–0.69) and moderate intra-reader reliability (mean kappa 0.55; range 0.28–0.81). With the atlas, inter-reader reliability improved in round 1 to moderate (mean kappa 0.52; range 0.31–0.77) and intra-reader reliability to good (mean kappa 0.69; range 0.46–0.86). Higher intra-reader reliability was noted in participants with previous SGUS experience. The SGUS atlas increased both intra- and inter-reader reliability for scoring gland pathology in participants with varying SGUS experience suggesting a possible future role in clinical practice and trials.

**Table:** 

**Key Points** • *Ultrasonography can detect parenchymal changes in salivary glands in patients with Sjögren’s disease.* • *An ultrasound atlas may improve reliability of scoring parenchymal changes in salivary glands.*

**Key Points**

• *Ultrasonography can detect parenchymal changes in salivary glands in patients with Sjögren’s disease.*

• *An ultrasound atlas may improve reliability of scoring parenchymal changes in salivary glands.*

## Introduction

Primary Sjögren’s syndrome (pSS) is a chronic autoimmune disease, characterized by dryness of the eyes and mouth, extra glandular symptoms such as fatigue, pain in muscles and joints, interstitial lung disease and presence of autoantibodies and high levels of immunoglobulins. The diagnosis of pSS is often based on the classification criteria provided by the American College of Rheumatology (ACR) and the European League Against Rheumatism (EULAR) consensus criteria from 2016 [1].

Though not a part of the classification criteria, ultrasonography has a diagnostic value in pSS [2, 3]. The most frequent ultrasonographic features of pSS in the salivary glands are inhomogeneity of the gland, anechoic/hypoechoic areas, and fibrotic tissue. Ultrasonography of the salivary glands (SGUS) can easily be performed within 15 min and may be helpful in evaluating the pSS disease severity and morphological changes in the glands [4, 5].

Recently, the Outcome Measures in Rheumatology (OMERACT) ultrasound working group has validated ultrasound definitions of elementary lesions in major salivary glands in patients with pSS as well as a gray-scale consensus-based semi-quantitative scoring system (0–3) to facilitate use in clinical practice and in clinical trials [3, 6].

The validated gray-scale scoring system has been published with a written definition for each grade (0–3) with a corresponding imaging example for each grade [3, 6]. The reliability of this new scoring system has been tested by experts of the OMERACT ultrasound working group in stored video-clips with excellent intra-reader and good inter-reader agreement [3]. However, when tested in patients the reliability was lower and the agreement only moderate [6]. The use of an ultrasound atlas could potentially be a useful tool for improving reliability when scoring SGUS pathology, as use of an ultrasound atlas has previously been demonstrated to have a positive effect on the intra- and inter-reader reliability among experts when scoring synovitis [7].

An SGUS atlas has already been developed based on the OMERACT-SGUS semi-quantitative scoring system [2] and the objective of this pilot study was to assess the impact of using the SGUS atlas on the reliability of scoring salivary gland pathology. This was determined by assessing the intra- and inter-reader reliability of the OMERACT SGUS scoring system in participants with varying SGUS experience. We applied the written definition of the scoring system—as provided by the OMERACT group—and subsequently a recently published SGUS atlas based on the written OMERACT SGUS scoring system. The impact of the SGUS atlas as compared to the written definition alone was assessed [2].

## Methods

### Participants

Nine rheumatologists and one medical student participated in the pilot study. The medical student had 1 year of experience solely with SGUS. All nine rheumatologists were trained in musculoskeletal ultrasound (MSUS) with >5 years of experience but with varying experience in SGUS (none to >5 years of experience).

### Images for the reliability exercise

SGUS images were collected by the facilitator (LT) who participated in the OMERACT ultrasound working group that developed the SGUS scoring system. The images consisted of 30 gray-scale US images of the salivary glands, the number of images in line with previous OMERACT exercises in static images [8]. All the images were collected from patients referred to the Center for Rheumatology and Spine Diseases, Rigshospitalet, Glostrup, Denmark, for possible Sjögren’s disease.

**Fig. 1:**
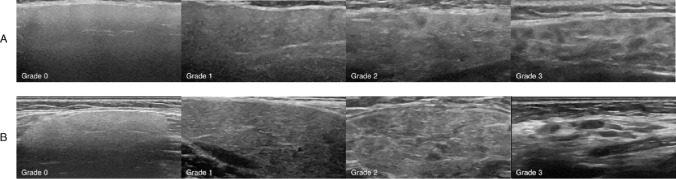
SGUS atlas. Ultrasound images examples of the four-grade semi-quantitative scoring system developed and validated by the OMERACT ultrasound working group: parotid gland (**A**) and submandibular gland (**B**) grade 0, normal parenchyma; grade 1, minimal change: mild inhomogeneity without anechoic/hypoechoic areas; grade 2, moderate change: moderate inhomogeneity with focal anechoic/hypoechoic areas; grade 3, severe change: diffuse inhomogeneity with anechoic/hypoechoic areas occupying the entire gland surface

Fourteen images of the parotid gland (PG) and 16 images of the submandibular glands (SMG) from 30 different individuals with or without pSS were included. The images displayed different grades of pathology (0–3) representing differences in severity of disease damage. Examples of grade 0–3 for both PG and SMG are illustrated in Fig. 1. All the images were obtained using a GE Logiq® E9 R5 (Milwaukee, WI, USA) ultrasound machine with a 5-16ML linear array transducer. Any identifiers on the images were removed.

### Scoring system and SGUS atlas

The newly developed and validated OMERACT semi-quantitative gray-scale scoring system for pSS (0–3) was applied in the study [2]. The scores are defined as follows: grade 0 = normal parenchyma; grade 1 = mild inhomogeneity without anechoic or hypoechoic areas and hyperechogenic bands; grade 2 = moderate inhomogeneity with focal anechoic or hypoechoic areas; and grade 3 = severe inhomogeneity with diffuse an- or hypoechoic areas occupying the entire gland or a fibrous gland [5]. Based on these definitions and imaging examples, an atlas had previously been developed by one of the ultrasonographers with longstanding experience in SGUS (VF) [2]. The atlas consisted of 4 different imaging examples for every grade 0–3 for both the SMG and for the PG—see Fig. 1.

### Reliability exercise

Using a web-based exercise, the 10 participants scored all images utilizing the OMERACT scoring system for pSS first using the written definitions and subsequently using the SGUS atlas. To assess intra-reader reliability for the written definition and the atlas, two rounds for each method were conducted with a 4-week interval between rounds. Between rounds the images were rearranged in a random order.

### Statistical analysis

The intra- and inter-reader reliability of scoring images were assessed on static images according to kappa (κ) analyses [9]. Intra-reader reliability on 30 images was assessed using Cohen’s kappa between the two rounds of scoring for each expert for the written definitions and for the SGUS atlas. A mean Cohen’s kappa was determined for all scorings. For inter-reader reliability, Cohen’s kappa was computed between all readers on 30 images for each round of scoring. A mean Cohen’s kappa for each round was determined.

Furthermore, percentage of exact agreement (PEA) and percentage of close agreement (PCA) were calculated for scoring the glands. The PCA was defined as the percentage of the patients where the scores differed by no more than 1.

Kappa values were interpreted according to Landis and Koch as kappa values of 0–0.2 were considered as poor, 0.21–0.40 fair, 0.41–0.60 moderate, 0.61–0.80 good, and 0.81–1 excellent agreement [10].

All analyses were performed using R (R 4.2.2 GUI 1.79 High Sierra build (8160)).

## Results

### Reliability of scoring salivary gland pathology using the written scoring system

The results of the intra-reader reliability are shown in Table 1 together with the SGUS experience of the participants. Intra-reader reliability showed moderate agreement with a mean kappa of 0.55 (range 0.28–0.81), mean PEA was 67% (range 47–87%), and mean PCA was 99% (range 93–100%).

**Table 1:** Intra-reader reliability in Cohen’s kappa and percentage exact and close agreement

Raters	SGUS experience (years)	1. scoring without atlas vs 2. scoring without atlas		1. scoring with atlas vs 2. scoring with atlas	
		Kappa	PEA/PCA (%/%)	Kappa	PEA/PCA (%/%)
Rater 1	>5	0.58	70/100	0.86	90/97
Rater 2	>5	0.55	67/100	0.64	73/100
Rater 3	2–3	0.63	73/97	0.68	73/100
Rater 4	1–2	0.50	63/100	0.68	76/100
Rater 5	1–2	0.69	77/100	0.86	87/100
Rater 6*	1	0.81	87/100	0.86	90/100
Rater 7	<1	0.50	63/100	0.57	70/97
Rater 8	<1	0.48	63/100	0.59	70/100
Rater 9	<1	0.47	60/93	0.73	80/100
Rater 10	<1	0.28	47/97	0.46	60/100
Mean		0.55	67/99	0.69	77/99

Kappa interpretation: 0–0.2 were considered as poor, 0.21–0.40 fair, 0.41–0.60 moderate, 0.61–0.80 good, and 0.81–1 excellent agreement. Percent of exact agreement (PEA) expresses the percentage of the patients receiving the same score and percent of close agreement (PCA) is the percentage of the patients where the scores differ no more than one

*Student with 12-month experience of regularly performing SGUS and scoring with the written definitions

The inter-reader reliability without the atlas had fair agreement in the first round with a mean kappa of 0.36 (range 0.06–0.69), mean PEA of 53% (range 37–77%), and mean PCA of 96% (range 87–100%). In the second round, the agreement was moderate with mean kappa of 0.46 (range 0.19–0.73), mean PEA of 60% (range 37–80%), and mean PCA of 96% (range 83–100%). The inter-observer reliability stratified by SGUS experience including the PEA/PCA is shown in Table 2.

**Table 2:** Inter-reader reliability between readers, Cohen’s kappa above the diagonal and percentage of exact agreement (PEA, %)/percentage of close agreement (PCA, %) under the diagonal

1. Round without atlas	SGUS experience	Reader 1	Reader 2	Reader 3	Reader 4	Reader 5	Reader 6	Reader 7	Reader 8	Reader 9	Reader 10
Reader 1	>5	1	0.37 (53/100)	0.38 (53/100)	0.33 (50/93)	0.37 (53/97)	0.45 (60/97)	0.37 (53/97)	0.50 (63/100)	0.34 (50/100)	0.20 (40/93)
Reader 2	>5	0.37 (53/100)	1	0.46 (60/100)	0.32 (50/100)	0.38 (53/97)	0.24 (43/100)	0.51 (63/100)	0.15 (37/93)	0.69 (77/100)	0.32 (50/97)
Reader 3	2–3	0.38 (53/100)	0.46 (60/100)	1	0.52 (67/97)	0.29 (47/90)	0.18 (37/93)	0.45 (60/97)	0.24 (43/93)	0.47 (60/97)	0.45 (63/90)
Reader 4	1–2	0.33 (50/93)	0.32 (50/100)	0.52 (67/97)	1	0.42 (57/90)	0.30 (47/87)	0.36 (53/100)	0.29 (47/87)	0.38 (53/100)	0.32 (53/97)
Reader 5	1–2	0.37 (53/97)	0.38 (53/97)	0.29 (47/90)	0.42 (57/90)	1	0.35 (53/100)	0.59 (70/97)	0.45 (60/93)	0.39 (53/93)	0.32 (50/93)
Reader 6	1	0.45 (60/97)	0.24 (43/100)	0.18 (37/93)	0.30 (47/87)	0.35 (53/100)	1	0.36 (53/100)	0.39 (57/97)	0.31 (47/100)	0.06 (30/100)
Reader 7	<1	0.37 (53/97)	0.51 (63/100)	0.45 (60/97)	0.36 (53/100)	0.59 (70/97)	0.36 (53/100)	1	0.36 (53/93)	0.43 (57/97)	0.43 (60/97)
Reader 8	<1	0.50 (63/100)	0.15 (37/93)	0.24 (43/93)	0.29 (47/87)	0.45 (60/93)	0.39 (57/97)	0.36 (53/93)	1	0.35 (50/90)	0.27 (47/90)
Reader 9	<1	0.34 (50/100)	0.69 (77/100)	0.47 (60/97)	0.38 (53/100)	0.39 (53/93)	0.31 (47/100)	0.43 (57/97)	0.35 (50/90)	1	0.30 (47/97)
Reader 10	<1	0.20 (40/93)	0.32 (50/97)	0.45 (63/90)	0.32 (53/97)	0.32 (50/93)	0.06 (30/100)	0.43 (60/97)	0.27 (47/90)	0.30 (47/97)	1
2. Round without atlas	SGUS experience	Reader 1	Reader 2	Reader 3	Reader 4	Reader 5	Reader 6	Reader 7	Reader 8	Reader 9	Reader 10
Reader 1	>5	1	0.32 (47/97)	0.61 (70/97)	0.39 (53/83)	0.47 (60/97)	0.63 (73/100)	0.39 (57/100)	0.29 (47/97)	0.47 (60/100)	0.46 (60/100)
Reader 2	>5	0.32 (47/97)	1	0.46 (60/97)	0.37 (53/100)	0.60 (70/97)	0.47 (60/97)	0.47 (60/100)	0.46 (60/93)	0.39 (53/97)	0.56 (67/97)
Reader 3	2–3	0.61 (70/97)	0.46 (60/97)	1	0.60 (70/93)	0.47 (60/97)	0.47 (60/93)	0.46 (60/97)	0.45 (60/97)	0.48 (60/93)	0.51 (63/97)
Reader 4	1–2	0.39 (53/83)	0.37 (53/100)	0.60 (70/93)	1	0.30 (47/93)	0.32 (47/83)	0.52 (63/83)	0.19 (37/97)	0.39 (50/80)	0.35 (50/93)
Reader 5	1–2	0.47 (60/97)	0.60 (70/97)	0.47 (60/97)	0.30 (47/93)	1	0.55 (67/97)	0.56 (67/100)	0.50 (63/97)	0.51 (63/100)	0.73 (80/97)
Reader 6	1	0.63 (73/100)	0.47 (60/97)	0.47 (60/93)	0.32 (47/83)	0.55 (67/97)	1	0.59 (70/100)	0.45 (60/97)	0.55 (67/100)	0.55 (67/100)
Reader 7	<1	0.39 (57/100)	0.47 (60/100)	0.46 (60/97)	0.52 (63/83)	0.56 (67/100)	0.59 (70/100)	1	0.31 (50/97)	0.43 (57/100)	0.50 (63/100)
Reader 8	<1	0.29 (47/97)	0.46 (60/93)	0.45 (60/97)	0.19 (37/97)	0.50 (63/97)	0.45 (60/97)	0.31 (50/97)	1	0.31 (47/97)	0.45 (60/97)
Reader 9	<1	0.47 (60/100)	0.39 (53/97)	0.48 (60/93)	0.39 (50/80)	0.51 (63/100)	0.55 (67/100)	0.43 (57/100)	0.31 (47/97)	1	0.55 (67/100)
Reader 10	<1	0.46 (60/100)	0.56 (67/97)	0.51 (63/97)	0.35 (50/93)	0.73 (80/97)	0.55 (67/100)	0.50 (63/100)	0.45 (60/97)	0.55 (67/100)	1
1. Round with atlas	SGUS experience	Reader 1	Reader 2	Reader 3	Reader 4	Reader 5	Reader 6	Reader 7	Reader 8	Reader 9	Reader 10
Reader 1	>5	1	0.37 (53/97)	0.68 (77/100)	0.47 (60/87)	0.42 (57/97)	0.77 (83/97)	0.36 (53/93)	0.54 (67/100)	0.64 (73/97)	0.31 (50/97)
Reader 2	>5	0.37 (53/97)	1	0.55 (67/100)	0.46 (60/97)	0.69 (70/100)	0.59 (70/100)	0.45 (60/97)	0.59 (70/100)	0.46 (60/100)	0.41 (57/100)
Reader 3	2–3	0.68 (77/100)	0.55 (67/100)	1	0.57 (67/90)	0.59 (70/100)	0.76 (83/100)	0.44 (60/97)	0.58 (70/100)	0.73 (80/100)	0.41 (57/100)
Reader 4	1–2	0.47 (60/87)	0.46 (60/97)	0.57 (67/90)	1	0.47 (60/100)	0.53 (63/90)	0.51 (63/97)	0.47 (60/93)	0.65 (73/90)	0.42 (57/97)
Reader 5	1–2	0.42 (57/97)	0.69 (70/100)	0.59 (70/100)	0.47 (60/100)	1	0.55 (67/100)	0.55 (67/100)	0.46 (60/100)	0.51 (63/100)	0.46 (60/100)
Reader 6	1	0.77 (83/97)	0.59 (70/100)	0.76 (83/100)	0.53 (63/90)	0.55 (67/100)	1	0.58 (70/97)	0.53 (67/100)	0.68 (77/100)	0.35 (53/100)
Reader 7	<1	0.36 (53/93)	0.45 (60/97)	0.44 (60/97)	0.51 (63/97)	0.55 (67/100)	0.58 (70/97)	1	0.49 (63/97)	0.46 (60/97)	0.45 (60/100)
Reader 8	<1	0.54 (67/100)	0.59 (70/100)	0.58 (70/100)	0.47 (60/93)	0.46 (60/100)	0.53 (67/100)	0.49 (63/97)	1	0.50 (63/100)	0.54 (67/100)
Reader 9	<1	0.64 (73/97)	0.46 (60/100)	0.73 (80/100)	0.65 (73/90)	0.51 (63/100)	0.68 (77/100)	0.46 (60/97)	0.50 (63/100)	1	0.55 (67/97)
Reader 10	<1	0.31 (50/97)	0.41 (57/100)	0.41 (57/100)	0.42 (57/97)	0.46 (60/100)	0.35 (53/100)	0.45 (60/100)	0.54 (67/100)	0.55 (67/97)	1
2. Round with atlas	SGUS experience	Reader 1	Reader 2	Reader 3	Reader 4	Reader 5	Reader 6	Reader 7	Reader 8	Reader 9	Reader 10
Reader 1	>5	1	0.51 (63/100)	0.40 (57/97)	0.40 (53/97)	0.54 (67/100)	0.76 (83/100)	0.71 (80/100)	0.57 (70/100)	0.43 (57/100)	0.45 (60/100)
Reader 2	>5	0.51 (63/100)	1	0.64 (73/97)	0.55 (67/100)	0.69 (77/100)	0.64 (73/100)	0.51 (63/100)	0.46 (60/100)	0.73 (80/100)	0.60 (70/100)
Reader 3	2–3	0.40 (57/97)	0.64 (73/97)	1	0.47 (60/97)	0.68 (77/97)	0.54 (67/97)	0.57 (70/97)	0.45 (60/97)	0.55 (67/97)	0.63 (73/100)
Reader 4	1–2	0.40 (53/97)	0.55 (67/100)	0.47 (60/97)	1	0.56 (67/97)	0.27 (43/97)	0.29 (43/93)	0.41 (53/93)	0.73 (80/100)	0.56 (67/97)
Reader 5	1–2	0.54 (67/100)	0.69 (77/100)	0.68 (77/97)	0.56 (67/97)	1	0.59 (70/100)	0.54 (67/100)	0.59 (70/100)	0.60 (70/100)	0.82 (87/100)
Reader 6	1	0.76 (83/100)	0.64 (73/100)	0.54 (67/97)	0.27 (43/97)	0.59 (70/100)	1	0.85 (90/100)	0.62 (73/100)	0.38 (53/100)	0.50 (63/100)
Reader 7	<1	0.71 (80/100)	0.51 (63/100)	0.57 (70/97)	0.29 (43/93)	0.54 (67/100)	0.85 (90/100)	1	0.66 (77/100)	0.34 (50/100)	0.44 (60/100)
Reader 8	<1	0.57 (70/100)	0.46 (60/100)	0.45 (60/97)	0.41 (53/93)	0.59 (70/100)	0.62 (73/100)	0.66 (77/100)	1	0.43 (57/97)	0.59 (70/100)
Reader 9	<1	0.43 (57/100)	0.73 (80/100)	0.55 (67/97)	0.73 (80/100)	0.60 (70/100)	0.38 (53/100)	0.34 (50/100)	0.43 (57/97)	1	0.60 (70/100)
Reader 10	<1	0.45 (60/100)	0.60 (70/100)	0.63 (73/100)	0.56 (67/97)	0.82 (87/100)	0.50 (63/100)	0.44 (60/100)	0.59 (70/100)	0.60 (70/100)	1

### Reliability of scoring salivary gland pathology using the SGUS atlas

Intra-reader reliability using the SGUS atlas was good with a mean kappa of 0.69 (range 0.46–0.86), mean PEA of 77% (range 60–90%), and mean PCA of 99% (range 97–100%).

The inter-reader reliability of the SGUS atlas was moderate in both the first and second round with a mean kappa of respectively 0.52 (range 0.31–0.77) and 0.54 (range 0.27–0.85), mean PEA of 65% (range 50–83%) to 67% (range 43–90%), and mean PCA of 96% (range 87–100%) to 99% (range 93–100%).

### Impact of using the SGUS atlas

The intra-reader reliability improved from moderate without the atlas to good when using the atlas—from mean kappa of 0.55 to 0.69 and mean PEA of 67 to 77%. Mean PCA was 99% with and without the atlas. The inter-reader reliability improved from fair without the atlas to moderate using the atlas in the 1st round—from a mean kappa 0.36 to 0.52 and mean PEA of 53 to 65%. Mean PCA was 96% with and without the atlas in 1st round. In the 2nd round of scoring, the agreement was moderate with increasing mean kappa from 0.46 without the atlas to 0.54 with the atlas. The mean PEA improved from 60% without the atlas to 67% with the atlas and mean PCA from 96 to 99% with the atlas.

The SGUS experience also impacts the intra-reader reliability when using the atlas (Table 1) with highest kappa values for the medical student and those with the longest SGUS experience though all readers improved.

## Discussion

In this pilot study, we assessed the impact of an SGUS atlas on the reliability of the new OMERACT SGUS scoring system by testing the reliability with and without the use of an SGUS atlas among participants with varying experience in performing SGUS. We found that the use of the SGUS atlas improved intra-reader reliability and PEA for scoring gland pathology compared to the written definitions. The SGUS atlas also improved inter-reader reliability and PEA in round 1 compared with the written definitions which was further established in round 2. The fact that inter-reader reliability improved between round 1 and 2 when scoring with the atlas indicate that even minimal training has an impact, and that the reliability could potentially improve further with additional training. The use of an SGUS atlas may be important for clinical trials and a useful clinical tool in routine care as grades ≥ 2 in at least one gland have a high specificity for the diagnosis of pSS with a high positive predictive value [2, 11–14].

Reliability in the current pilot study was lower than the reported reliability among experts assessing video-clips or static images [3, 15]. This discrepancy may partly be the differences in SGUS training between the participants where some had very limited experience whereas the participants in the other studies had a minimum of 2-year experience with SGUS. As seen in Table 1, considerably higher kappa scores were obtained in participants with previous experience with SGUS than among novices. This indicates that some basic experience with scanning and assessing salivary glands is a necessity for reliably scoring pathology in patients when using an atlas for support. The reliability assessment in a recent study suggests that the impact of SGUS experience disappears among participants who all have a certain knowledge and training in SGUS [15]. Further, it is of importance that inter-reader reliability can be improved by calibration between readers, reflected by wide variation of kappa and PEA between readers but a consistent PCA, as shown in Table 2. Practically, it is seen that the reliability is increasing with use of an atlas in the second round, giving more consistent scorings between readers and to some extent compensating for SGUS experience.

The impact of the SGUS atlas on the reliability was lower than what has previously been reported when using an ultrasound atlas for scoring synovitis [7]. However, most rheumatologists who use ultrasonography in clinical practice are more experienced with MSUS than SGUS where an atlas for scoring synovitis may add to a competency already existing as compared to scoring salivary gland pathology. To assess the possible impact of MSUS experience in learning SGUS scoring, a study would be needed that include ultrasound novices.

Our pilot study suggests that using an SGUS atlas may provide a valuable tool for improving the reliability when scoring damage in the major salivary glands in patients with pSS. The atlas, together with training, may contribute to a more homogenous scoring of gland pathology in pSS in routine clinical practice and in clinical trials.

The pilot study has limitations. We used 30 images for the reliability exercise. This may be a small number but is in line with previous OMERACT reliability exercises in static images [8]. Additionally, we did not group the participants by SGUS experience as in the reliability exercises from the OMERACT group [15]. This could potentially explain the lower reliability found in our study as grouping the participants would homogenize the group and possibly enhance consistency within the group having the same levels of experience, training, and background knowledge. However, including participants with varying SGUS experience may also be a strength of the study as it resembles the use in routine care and supports the applicability across skills and thereby the external validity of the study.

Nevertheless, the reliability should also be tested in a patient-based exercise, and it is possible that the atlas in such a setting may be more valuable for rheumatologists with less practical SGUS skills. However, this first step indicates a value of the SGUS atlas and the role in training and routine care needs to be established.

In conclusion, we found that the SGUS atlas improved the reliability of scoring salivary glands in patients with pSS and resulted in moderate to excellent intra-reader reliability depending on the SGUS experience and a moderate inter-reader reliability. The reliability of the atlas needs to be tested in patients.
